# Synovial membrane immunohistology in early-untreated rheumatoid arthritis reveals high expression of catabolic bone markers that is modulated by methotrexate

**DOI:** 10.1186/ar4398

**Published:** 2013-12-03

**Authors:** Shankar Revu, Petra Neregård, Erik af Klint, Marina Korotkova, Anca Irinel Catrina

**Affiliations:** 1 Rheumatology Unit, Department of Medicine, Karolinska University Hospital and Karolinska Institutet, S-17176 Stockholm, Sweden; 2 Actar AB, S-17165 Stockholm, Sweden

## Abstract

**Introduction:**

We aimed to investigate the expression and therapeutic modulation of the receptor activator of the NF-κB ligand (RANKL) system in early-untreated rheumatoid arthritis (RA).

**Methods:**

In this study, 15 patients with newly diagnosed RA (median symptom duration 7 months) were started on methotrexate (MTX) 20 mg weekly. Synovial biopsies were obtained by needle arthroscopy at baseline and 8 weeks after initiation of therapy. X-rays of the hands and feet were obtained at baseline and 1 year after diagnosis. Immunohistochemistry was performed to detect RANKL, receptor activator of nuclear factor-κB (RANK) and osteoprotegerin (OPG) in the synovial biopsies. The *in vitro* effect of MTX was tested on RA-derived primary fibroblasts and the osteoblasts-like osteosarcoma cell line (rtPCR, Western blot and ELISA) and in osteoclasts (tartrate-resistant acid phosphatase staining and dentine pit formation assay).

**Results:**

MTX decreased synovial cellularity as well as RANK expression and the RANKL/OPG ratio. We confirmed this effect by a decrease of the mRNA and protein RANKL/OPG ratio in synovial-derived fibroblasts and osteoblasts-like tumoral cells exposed *in vitro* to methotrexate. Supernatants from MTX treated osteoblasts-like tumoral cells prevented pre-osteoclast formation in the absence of exogenous RANKL. Furthermore, MTX blocked osteoclastogenesis from peripheral blood mononuclear cells despite the presence of macrophage colony stimulating factor and RANKL, which indicates that MTX directly inhibits osteoclastogenesis.

**Conclusions:**

The synovial membrane of early-untreated RA is characterized by a high RANKL/OPG ratio that can be reversed by methotrexate.

## Introduction

Rheumatoid arthritis (RA) is a chronic inflammatory disease characterized by synovial inflammation and bone destruction in the absence of early and aggressive therapeutic intervention [1]. Bone destruction is controlled by the complex interplay between three molecules essential for bone biology: a receptor (receptor activator of NF-κB (RANK)), a ligand (receptor activator of the NF-κB ligand (RANKL)) and a soluble decoy receptor (osteoprotegerin (OPG)) [2]. In RA it has been postulated that bone destruction occurs as a direct consequence of inflammation. This has been challenged recently by the observation that bone destruction can occur in RA despite clinical remission [3,4], while specific anti-rheumatic drugs might protect against destruction even in the absence of clinical remission [5,6]. Taken together these findings suggest that inflammation and bone destruction are at least partially uncoupled in RA.

RANKL, RANK and OPG are all expressed in the inflamed synovium of RA and specifically modulated by distinct anti-rheumatic drugs. We have previously shown that anti-tumor necrosis factor (TNF) agents and glucocorticoids directly modulate the RANKL system in human RA [7,8]. More recent animal studies have demonstrated a bone-protective effect of methotrexate (MTX) [9,10], abatacept [11] and tocilizumab [12]. To date no study to characterize the RANKL system in early-untreated RA or its modulation by MTX is currently available. We therefore aimed to characterize RANKL, RANK and OPG expression in the synovial membrane of early-untreated RA in relation to local inflammation and disease activity and to investigate how expression of these molecules might change following MTX treatment.

## Methods

### Patients

Fifteen patients (10 women and five men, median age 56 years, range 33 to 78 years) with new diagnosed RA (patient-reported symptom duration <1 year, median symptom duration 7 months, range 2 to 12 months) according to 1987 American College of Rheumatology criteria [13], previously disease-modifying anti-rheumatic drug naïve, were started on MTX 10 mg weekly and reached a stable dose of 20 mg after 2 weeks of increasing the dose by 5 mg each week. Synovial biopsy samples were obtained by arthroscopy from all patients before and after a median of 8 weeks of treatment. Clinical evaluation and evaluation of the therapeutic response according to European League Against Rheumatism (EULAR) response criteria (a median of 3 months after MTX initiation) were performed by a rheumatologist. X-ray scans of hands and feet were obtained at baseline and 1 year after diagnosis. Nonsteroidal anti-inflammatory drugs, per oral prednisolone to a maximum dose of 10 mg daily and intraarticular glucocorticoid injections in joints other than the one subjected to biopsy were permitted according to clinical indication. Additional file 1 summarizes the demographic characteristics of the patients. All procedures where approved by the Northern Stockholm Ethical Review Board and informed consent was obtained from all the participants in the study.

### Immunohistochemistry evaluation

Presence of RANKL, RANK and OPG was detected using the following monoclonal antibodies: anti-human RANKL antibody at a final concentration of 5 μg/ml (12A668; Abcam, Cambridge, UK), anti-human RANK antibody at a final concentration of 5 μg/ml (9A725; Abcam) and anti-human OPG antibody (MAB805; R&D Systems, Minneapolis, MN, USA) at a concentration of 2.5 μg/ml, using a previously published protocol [7]. We further characterized the synovial cell phenotype using several monoclonal antibodies: anti-human CD3 antibody (347340; BD Biosciences San Jose, CA, USA), anti-human CD68 antibody (M0814; DakoCytomation, Glostrup, Denmark), anti-human CD31 antibody (clone EN4, MON6002; Novakemi Ab, Uden, the Netherlands), anti-human CD138 antibody (B-A38; Diaclone Res, Besancon, France) and anti-human CD19 antibody (HD37; DakoCytomation). The isotype of all primary antibodies was IgG1 and a concentration-matched mouse IgG1 was used as the negative control (DakoCytomation).

Synovial biopsy sections were evaluated semiquantitatively, using a four-point scale (0 = no staining, 1 = low amounts of staining, 2 = moderate amounts of staining, 3 = high amounts of staining) by two independent observers (SR and AIC) who were unaware of the patient’s identity and biopsy sequence, as described previously [8]. Final scores represent the means of the two independent observations. For quantification, synovial expression of three of the stained markers (RANKL, RANK and OPG) was evaluated using computer-assisted image analysis (250× magnifications) as described previously [7].

### Cell culture and western blot analysis

The human osteoblast-like sarcoma cell line (SaOS_2_) was purchased from DSMZ (Braunschweig, Germany), and cultured in McCoy’s 5A medium (Invitrogen, Carlsbad, CA, USA). RA synovial-derived fibroblasts from two different donors were purchased from Dominion Pharmakine (Deiro, Bizkaia, Spain) and cultured in Dulbecco’s modified Eagle’s medium. All media were supplemented with 10% heat-inactivated fetal bovine serum. All reagents were purchased from Gibco (Carlsbad, CA, USA).

Cells were seeded at a density of 10^7^ cells/ml on 10 cm dishes at 37°C in a humidified atmosphere with 5% carbon dioxide and incubated with or without MTX at a final concentration of 1, 10 or 100 μM. After 24 hours cells were washed with cold phosphate-buffered saline (PBS) and whole cell lysates were prepared with RIPA buffer containing 50 mM Tris HCl, pH 7.5, 150 mM NaCl, Igepal CA-630 (all reagents from Sigma, Stockholm, Sweden), and complete protease inhibitor cocktail tablet (Roche, Basel, Switzerland). Lysed cells were centrifuged to remove debris at 14,000 rpm for 15 minutes at 4°C and supernatants were collected. A total of 40 μg cellular protein (measured using the Bradford assay and bovine serum albumin as standards) was loaded into sodium dodecyl sulphate polyacrylamide gel electrophoresis under nonreducing conditions. Following electrophoresis, proteins were transferred on to a Polyvinylidene difluoride (PVDF) membrane and blocked with 5% nonfat milk in 0.1% PBS/Tween 20 for 1 hour at room temperature. The membrane was incubated with either anti-human RANKL or anti-human OPG mouse monoclonal antibodies at a final concentration of 2 μg/ml in 0.1% PBS/Tween 20 at 4°C overnight. After washing, the membrane was incubated with secondary peroxidase-linked anti-mouse IgG (NXA931; Amersham Biosciences, Buckinghamshire, UK) diluted in 0.1% PBS/Tween 20 diluted 1:3,000 for 1 hour at room temperature. After washing the membrane, protein bands were visualized on X-ray film using enhanced chemiluminescence (RPN2209; Amersham Biosciences). For analyzing the protein expression, band intensity was measured by computer-assisted image analysis and normalized using β-actin. All experiments were run in triplicate and were repeated at least four times.

### Enzyme-linked immunosorbent assay

All culture supernatants were centrifuged and stored at -20°C for a short time with no thawing/freezing cycles until quantified. Samples were assayed using commercially available kits for RANKL (BI-202452) and OPG (BI-20402) using a sandwich enzyme immunoassay according to the manufacturer’s instructions (Biomedica, Vienna, Austria). All samples were analyzed as duplicates from at least three independent experiments. The concentration of the samples was calculated from the standard curve using a 4PL algorithm and expressed as picomoles per liter. All experiments were run in triplicate and were repeated at least four times.

### Quantitative reverse transcriptase-polymerase chain reaction

Total RNA was isolated from Saos2 cells using the RNeasy mini purification Kit (Qiagen, Hilden, Germany) and cDNA was obtained using the Super Script cDNA synthesis kit (Invitrogen). RANKL and β-actin mRNA expression was detected by Taqman Gene Expression Assay Hs00243522 and Hs99999903 respectively (Applied Biosystems, Carlsbard, CA, USA). The mRNA expression of other genes was detected using the Power SYBR Green PCR Master Mix (Applied Biosystems, Warrington, UK). Primer sequences are provided in Additional file 2. The amplification conditions used for all genes were 10 minutes at 95°C followed by two-step cycling polymerase chain reaction for 40 cycles at 95°C for 15 seconds and at 60°C for 1 minute. All experiments were run in triplicate and were repeated at least four times.

### Osteoclastogenesis and bone destruction assay

CD14^+^ cells were isolated from healthy donor peripheral blood mononuclear cells (PBMCs) using magnetically labeled CD14 microbeads (130-050-201; Miltenyi Biotec, Bergisch Gladbach, Germany). CD14^+^ cells were collected and seeded at a density of 0.5 × 10^5^ cells/ml in Dulbecco’s modified Eagle’s medium containing 10% heat-inactivated fetal bovine serum either in chamber slides (177445; Nalge Nunc, Rochester, NY, USA) or on synthetic bone discs (BD BioCoat Osteologic Bone cell culture system, 354610; Discovery Labware, San Jose, CA, USA). After 3 days the supernatant was removed and cells were incubated with conditioned supernatants from osteoblast-like cells previously incubated with or without MTX. Cells were further incubated for 14 days and the supernatant exchanged with new batches of the conditioned medium every 3 days in the presence of macrophage colony-stimulating factor (M-CSF). After 12 days, cells seeded on chamber slides were washed once with PBS and stained using a tartrate-resistant acid phosphatase (TRAP) staining kit according to the manufacturer’s instructions (387-A; Sigma Aldrich, Stockholm, Sweden). Pre-osteoclast formation was evaluated as the percentage of TRAP-positive mononuclear and binuclear stained cells out of the total number of cells.

Alternatively, CD14^+^ PBMCs were cultured in the presence of 25 ng/ml human M-CSF (216-MC; R&D) and 50 ng/ml human RANKL (390-TN-010; R&D) with or without MTX to a final concentration of 0, 1, 10 or 100 μM. During incubation, fresh medium with differentiation factors and MTX were changed every 3 days for chamber slide cultures and every 7 days for bone disc cultures. After 14 days, cells seeded on chamber slides were washed once with PBS and stained using a TRAP staining kit according to the manufacturer’s instructions (387-A; Sigma Aldrich). Osteoclast formation was evaluated as the percentage of TRAP-positive stained multinuclear cells (≥3 nuclei) and pre-osteoclast formation was evaluated as the percentage of TRAP-positive mononuclear and binuclear stained cells out of the total number of cells. After 21 days, supernatants from the bone disc cultures were removed and the disks washed twice in distilled water, followed by extensive washing with 6% chlorine water until the cells dislodge from the discs and a final washing step with distilled water. Resorption areas were examined under normal light microscope and pictures were analyzed using Image J and Photoshop.

### Survival assay

Osteoclast precursors, osteoblast-like tumoral cells, synovial fibroblasts and PBMC were cultured with or without MTX at a final concentration of 100 μM. After 7 days, adherent and nonadherent cells were collected by gentle scraping and analyzed by flow cytometry analysis using the TACS Annexin V-FITC apoptosis detection kit (R&D Systems). Apoptotic cells were defined as Annexin-V-positive cells and expressed as a percentage of the total number of cells.

### Statistical analysis

Statistical analysis was performed using the Wilcoxon test for comparison of paired samples, the Mann–Whitney test for comparison of independent samples and the Spearman test for correlations. No mathematical correction for multiple comparisons was applied. Differences between proportions were analyzed by Fisher’s exact test. *In vitro* experiments results were analyzed using one-way analysis of variance as appropriate and the Dunnett *post hoc* test. *P* <0.05 was considered statistically significant.

## Results

### Methotrexate prevents radiographic progression in some but not all RA patients, independent of the clinical therapeutic response

Treatment with MTX resulted in clinical improvement with a significant decrease of the 28-joint Disease Activity Score from a mean ± standard error of the mean of 5.6 ± 0.2 before treatment to 3.5 ± 0.3 at 2 months after treatment initiation (*P* <0.05). A majority of the patients (13/15, 86%) achieved EULAR criteria for response (either good responders 6/15, 40%; or moderate responders 7/15, 47%), while two patients (2/15, 13%) were nonresponders. Erosions of hands and/or feet were present in two out of 15 patients at baseline (13%) and in six out of 14 patients at 1-year follow-up (40%, one missing data). Development of new erosions was identified in five out of 14 patients (30%, one missing data) at 1-year follow-up. Among these five patients, one (20%) was a EULAR nonresponder to therapy and four (80%) were responders. Among patients with no progression at follow-up, one out of 10 (10%) were EULAR nonresponders and nine (90%) were EULAR responders.

### Methotrexate treatment reduces synovial inflammation

**Figure 1:**
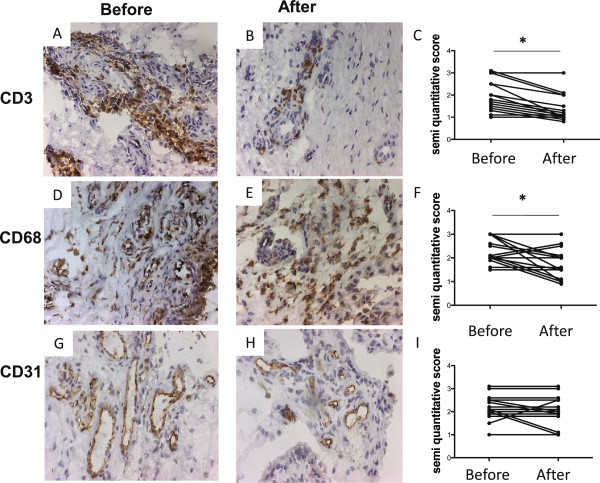
**Methotrexate decreases lymphocyte and macrophage infiltration in the rheumatoid synovium of early-untreated rheumatoid arthritis.** Brown immunohistochemistry (diaminobenzidine) staining of cell surface markers in synovial biopsies of early rheumatoid arthritis (RA). Synovial CD3 expression in early-untreated RA synovium **(A)** decreased significantly following 8 weeks of methotrexate (MTX) treatment **(B)**. Synovial CD68 expression in early, untreated RA synovium **(D)** decreased significantly following 8 weeks of MTX treatment **(E)**. Synovial CD31 expression in early, untreated RA **(G)** is not changed following 8 weeks of MTX treatment **(H)**. Results from the semi-quantitative double-blind analysis of synovial CD3 **(C)**, CD68 **(F)** and CD31 **(I)** expression before and 8 weeks after MTX treatment. *p<0.05.

Immunophenotyping of the synovial cells revealed a significant decrease of the lymphocyte CD3-positive population (from a median of 1.5 (range 1 to 3) to a median of 1 (range 1 to 3), *P* <0.05; Figure 1A,B,C) and the CD68 population (from a median of 2 (range 1.5 to 3) to a median of 1.5 (range 1 to 3), *P* <0.05; Figure 1D,E,F), with no changes in the expression of CD31 and in the synovial blood vessel size (*P* >0.05; Figure 1G,H,I). No changes in expression of CD19, CD138 or CD55 were observed (data not shown). No correlation between changes in synovial cell surface markers and changes in disease activity score was observed.

### Methotrexate significantly downregulates synovial expression of RANKL and RANK and decreases the RANKL/OPG ratio

**Figure 2:**
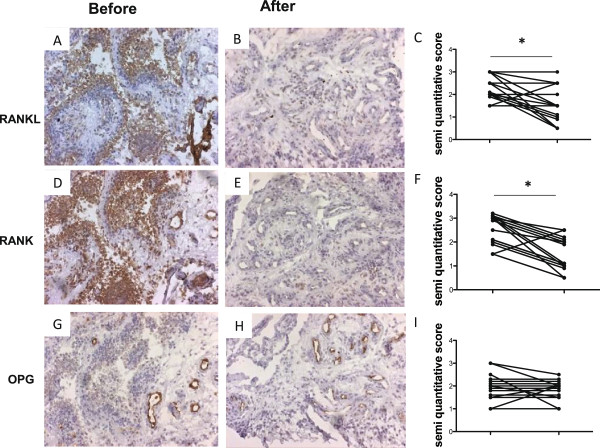
**Methotrexate decreases expression of RANKL and RANK in the rheumatoid synovium of early-untreated rheumatoid arthritis.** Brown immunohistochemistry (diaminobenzidine) staining of cell surface markers in synovial biopsies of early rheumatoid arthritis (RA). Synovial receptor activator of the NF-κB ligand (RANKL) expression in early, untreated RA synovium **(A)** decreased significantly following 8 weeks of methotrexate (MTX) treatment **(B)**, as evaluated by semi-quantitative double-blind microscopic analysis **(C)**. Synovial receptor activator of NF-κB (RANK) expression in early, untreated RA synovium **(D)** decreased significantly following 8 weeks of MTX treatment **(E)**, as evaluated by semi-quantitative double-blind microscopic analysis **(F)**. Synovial osteoprotegerin (OPG) expression in early, untreated RA **(G)** is not changed following 8 weeks **(H)** of MTX treatment, as evaluated by semi-quantitative double-blind microscopic analysis **(I)**. *p<0.05.

**Figure 3:**
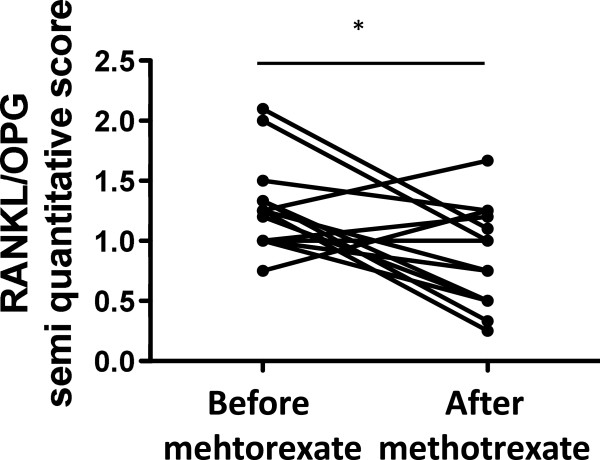
**Methotrexate decreases the synovial RANKL/OPG ratio in early-untreated rheumatoid arthritis.** Synovial receptor activator of the NF-κB ligand (RANKL)/osteoprotegerin (OPG) ratio as evaluated by semi-quantitative double-blind microscopic analysis. *P <0.05.

Changes in cellularity were accompanied by changes in local expression of RANKL with a significant decrease from a median score of 2 (range 1.5 to 3) to a median score of 1.5 (range 0.5 to 3) (*P* <0.05; Figure 2A,B,C) and RANK with a significant decrease from a median level of 3 (range 1.5 to 3) to a median score of 1 (range 0.5 to 2.5) (*P* <0.05; Figure 2D,E,F). No changes were observed in the expression of OPG (median score of 2 (range 1 to 3) before the treatment, and a median score of 2 (range 1 to 2.5) following treatment) (Figure 2G,H,I). These changes resulted in a significant decrease of the synovial RANKL/OPG ratio from a median of 1.25 (range 0.75 to 2) to a median of 0.75 (range 0.25 to 1.7) (*P* <0.05; Figure 3). Similar results were observed when the synovial RANKL/OPG ratio was analyzed by computer-assisted image analysis, showing a significant (*P* <0.05) decrease from a median of 2.6 (range 1.3 to 7.3) to a median of 1.8 (range 0.2 to 5.1). The synovial RANKL/OPG ratio at baseline was significantly higher (*P* <0.05) in patients with erosion progression at 1-year follow-up (median of 1.5, range 1 to 2) as compared with those with no progression (median 1.1, range 0.75 to 1.3). No correlation was observed between RANK, RANKL and OPG expression and CD marker expression.

### Methotrexate reduces RANKL expression *in vitro* in synovial-derived fibroblast cells and in osteoblast-like cells

**Figure 4:**
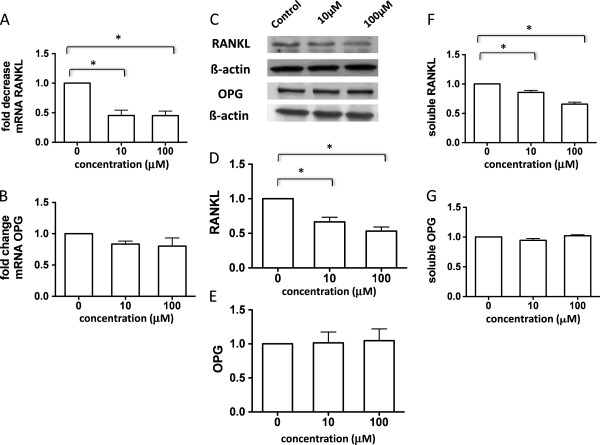
**Methotrexate decreases mRNA and protein expression of RANKL and RANK in rheumatoid arthritis synovial-derived fibroblasts.** A significant decrease in the receptor activator of the NF-κB ligand (RANKL) mRNA levels **(A)** with no changes in osteoprotegerin (OPG) mRNA levels **(B)** in the presence of methotrexate (MTX). Representative blots and graphs showing decrease of cellular RANKL protein expression **(C and D)** with no changes in the cellular OPG expression **(C and E)** in the presence of MTX. Decrease of soluble RANKL protein expression **(F)** with no changes in the soluble OPG expression **(G)** in the presence of MTX, where results are quantified by enzyme-linked immunosorbent assay. *p<0.05.

MTX reduced *in vitro* expression of mRNA RANKL expression in RA synovial-derived fibroblasts to 0.5 ± 0.08-fold of the control (*P* <0.05; Figure 4A), with no change in OPG mRNA expression (Figure 4B). A similar change was observed when cellular protein levels were analyzed by western blot (Figure 4C), with a decrease in RANKL expression up to 0.5 ± 0.1-fold of the control (*P* <0.05; Figure 4D) and no change in OPG expression (Figure 4E). Analysis of soluble proteins expression in the cell culture supernatants revealed a significant decrease of the RANKL levels to a maximum of 0.7 ± 0.03-fold of the control (*P* <0.05; Figure 4F), with no changes in the levels of soluble OPG (Figure 4G). Similar results were obtained in cultures of osteoblast-like tumoral cells, as shown in detail in Additional file 3. The MTX effect on RANKL expression was not mediated through cell death induction because incubation of either fibroblast-like or osteoblast-like cells with MTX failed to induce either necrosis or apoptosis (Additional file 4).

### Methotrexate decreases osteoclasts formation and bone resorption

**Figure 5:**
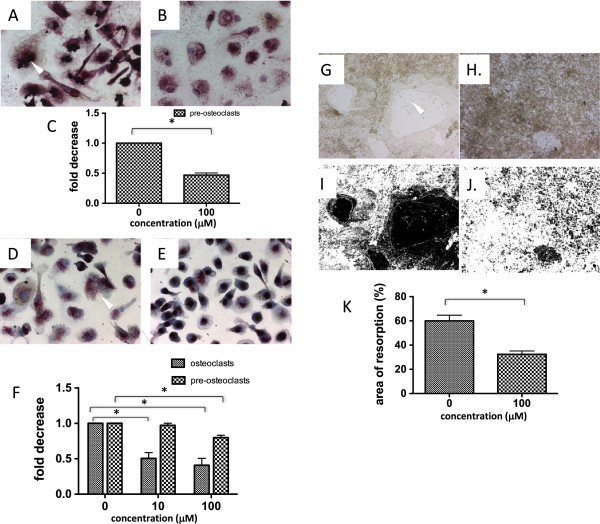
**Methotrexate suppresses osteoclastogenesis.** Tartrate-resistant acid phosphatase (TRAP) staining showing reduction in the number of TRAP-positive cells when CD14^+^ osteoclast precursors were incubated with supernatants from methotrexate (MTX)-treated osteoblast-like cells **(A)** as compared with supernatants from nontreated osteoblast-like cells **(B)**; arrow indicates a TRAP-positive pre-osteoclast. The fold decrease in the number of TRAP-positive pre-osteoclasts in MTX-treated osteoblast-like cells as compared with supernatants from nontreated osteoblast-like cells **(C)**. TRAP staining showed a reduction in the number of TRAP-positive cells when CD14^+^ osteoclast precursors were incubated with MTX **(D)** as compared with control **(E)** in the presence of exogenous macrophage colony-stimulating factor M-CSF and RANKL; arrow indicates a TRAP-positive multinucleated osteoclast. The fold decrease in the number of TRAP-positive pre-osteoclasts and TRAP-positive multinucleated (more than two nuclei) osteoclasts in MTX-treated cultures as compared with control cultures **(F)**. Von Kossa brown stained osteologic discs **(G)** showing large clear resorption areas (arrow) that are reduced following MTX exposure **(H)**. Inverted images with the black surface representing the bone resorption areas in the controls **(I)** and MTX-exposed cultures **(J)**, allowing quantification of data as seen in the adjacent graph **(K)**. *p<0.05.

Conditioned medium from osteoblast-like tumoral cells exposed to MTX reduced pre-osteoclast formation from CD14^+^ precursors in the presence of M-CSF alone (Figure 5A,B,C). Direct exposure of osteoclast precursors to MTX in presence of RANKL and M-CSF resulted in a significant decrease in the number of both pre-osteoclasts and mature osteoclasts to a maximum of 0.4 ± 0.1 of the control for pre-osteoclasts and 0.8 ± 0.03 for osteoclasts (Figure 5D,E,F). The decrease in osteoclast formation was paralleled by a significant reduction of synthetic bone resorption area from a mean ± standard error of the mean of 60% ± 4.7 in the controls to 32.4% ± 2.7 when MTX was added to the culture (Figure 5G,H,I,J,K). This effect was not mediated through cell death induction because incubation of osteoclasts precursors with MTX failed to induce either necrosis or apoptosis, as shown in Additional file 4.

## Discussion

MTX is the most common anti-rheumatic drug with a bone-sparing effect in some RA patients. We demonstrate here that MTX treatment modulates the RANKL/RANK/OPG pathway and inhibits osteoclasts maturation, two potential molecular mechanisms behind this clinical observed effect.

Our immunohistochemistry study is the first to investigate expression and modulation of the RANKL/RANK/OPG pathway in very early RA in disease-modifying anti-rheumatic drug-naïve patients. One earlier study in a heterogeneous RA population with median disease duration of 4 years and a large array of different anti-rheumatic treatments (including six patients only treated with MTX) suggested that successful disease-modifying anti-rheumatic drug treatment results in a significant decrease in the synovial RANKL/OPG ratio probably related to the decrease in inflammation. Our results, however, show that synovial RANKL and RANK expression are not completely dependent on local inflammation as demonstrated by the lack of association between these markers and synovial inflammation (evaluated by immunohistochemistry for CD3 and CD68). We have previously shown that intra-articular glucocorticoids decrease synovial expression of RANKL in the injected joint [8]. Intra-articular glucocorticoids in other joints than those that were subjected to arthroscopies were allowed in the current study according to clinical indication. Distant effects on the joint subjected to arthroscopy cannot be excluded, but even if present they should be minimal. The baseline synovial RANKL/OPG ratio was higher in patients showing radiographic progression during the first year of disease. This finding is in line with a previous study showing that the serum RANKL/OPG ratio is the best predictive marker for annual progression of radiological damage [14] independent of therapy. However, the small number of patients included in the current study precludes a definite conclusion and needs confirmation in a larger number of patients.

We further examined the effects of MTX *in vitro* in primary synovial-derived fibroblast and tumoral osteoblast-like cells. Long and repeated exposure *in vivo*, with local synovial and bone accumulation of MTX [15] and metabolites with vastly extended tissue half-lives [16], is challenging to reproduce *in vitro*. After extensive titration work, we chose to use doses that are higher than previous reported serum concentrations in RA patients [17,18] but are in the range of doses previously shown to be anti-inflammatory *in vitro*[19]. MTX decreases mRNA and protein RANKL expression without influencing OPG expression, in both synovial-derived fibroblasts and cultured osteoblast-like cells. We have previously demonstrated that TNF antagonists (such as etanercept and infliximab) are able to decrease the RANKL/OPG ratio through upregulation of synovial OPG expression [7]. We demonstrate here that MTX, similar to intra-articular corticosteroids but in contrast to TNF antagonists, modulates the synovial RANKL/OPG system through downregulation of RANKL. Taken together, our findings offer a rationale for the better effect observed following combination therapy with TNF antagonists and MTX than each treatment alone, with a potential dual-positive modulation of the RANKL/OPG system, resulting in better bone protection.

Previous reports demonstrated that MTX prevents osteoclastogenesis through decrease of RANKL expression [9,10]. We further demonstrate an additional direct effect of MTX on osteoclast precursor cells despite the presence of excess exogenous RANKL. Interestingly this effect is not modulated through cellular cytotoxicity as demonstrated by a lack of cell death induction in cells exposed to MTX. Previously, MTX was reported to suppress activation of the NF-κB signaling pathway [20], and NF-κB was reported essential for RANK-expressing osteoclast precursors to differentiate in TRAP-positive osteoclasts in response to RANKL and other osteoclastogenetic cytokines [21]. Further studies are needed to investigate whether MTX-induced suppression of the NF-κB signaling pathway could render osteoclasts precursors resistant to osteoclastogenesis.

In conclusion, we have demonstrated that untreated, early RA is characterized by an imbalanced synovial expression of RANKL, RANK and OPG independent of local inflammation. This could be reverted by MTX treatment in some but not all patients. MTX prevents osteoclastogenesis by a dual mechanism involving both reduction of RANKL expression and direct effects on osteoclast precursors independent of RANKL.

## Conclusions

Synovial membrane of early-untreated RA is characterized by a high RANKL/OPG ratio. Treatment with MTX directly affects the RANKL/RANK/OPG system and inhibits osteoclastogenesis in early, untreated RA.

## Abbreviations

EULAR: European League Against Rheumatism; M-CSF: Macrophage colony-stimulating factor; MTX: Methotrexate; NF-κB: Nuclear factor-kappa B; OPG: Osteoprotegerin; PBMC: Peripheral blood mononuclear cells; PBS: Phosphate-buffered saline; RA: Rheumatoid arthritis; RANK: Receptor activator of NF-κB; RANKL: Receptor activator of the NF-κB ligand; TNF: Tumor necrosis factor; TRAP: Tartrate-resistant acid phosphatase.

## Competing interests

The authors declare that they have no competing interests.

## Authors’ contributions

All authors made substantial contributions to acquisitions and analysis of the data. SR performed most of the experiments and participated in collection and analysis of data. PN recruited patients to the study and participated in analysis of immunohistochemistry data. EaK participated in the study design, recruited patients to the study and performed arthroscopies. MK participated in collection and analysis of the data. AIC designed the study, participated in collection and analysis of the data and drafted the manuscript. All authors critically revised the manuscript for important intellectual content, read and approved the final manuscript.

## Supplementary Material

Additional file 1 **Is a table listing detailed demographic information of the RA patients included in the study.** NSAID, nonsteroidal anti-inflammatory drugs; DAS, disease activity score; SEM, standard error of the mean; ACPA, anti-citrullinated protein antibodies; RF, rheumatoid factor. Click here for file

Additional file 2 **Presents supplementary methodological information.** Description of the RANKL and OPG primer sequences. Click here for file

Additional file 3 **Shows that MTX decreases mRNA and protein expression of RANKL and RANK in osteoblast-like tumoral cells.** Graph showing a significant decrease in the RANKL mRNA levels with no changes in OPG mRNA levels in the presence of MTX (A). Representative blot and graphs showing decrease of cellular RANKL protein expression with no changes in the cellular OPG expression in the presence of MTX (B). Graphs showing decrease of soluble RANKL protein expression with no changes in the soluble OPG expression in the presence of MTX (C), where results are quantified by enzyme-linked immunosobent assay (ELISA). rtPCR, reverse transcriptase polymerase chain reaction. Click here for file

Additional file 4 **Shows the lack of apoptosis induction by MTX.** Representative cytometry plots showing that MTX does not induce apoptosis and graphs representing quantification of Annexin-V-positive cells following MTX exposure in synovial fluid mononuclear cells (SFMC), PBMC, Saos-2 (osteoblast-like tumoral cells) and synovial fibroblasts. Click here for file
