# Supplementary material for: Synovial membrane immunohistology in early-untreated rheumatoid arthritis reveals high expression of catabolic bone markers that is modulated by methotrexate
Source: Arthritis Res Ther. 2013 Dec 3;15(6):R205. doi: 10.1186/ar4398 (PMC3978873; doi:10.1186/ar4398)
Supplement: Additional file 1 — Is a table listing detailed demographic information of the RA patients included in the study. NSAID, nonsteroidal anti-inflammatory drugs; DAS, disease activity score; SEM, standard error of the mean; ACPA, anti-citrullinated protein antibodies; RF, rheumatoid factor. [file ar4398-S1.doc]

|  | Patients demographic (n=15) |
| --- | --- |
| Median age (range) | 56 (33-78) |
| Gender | 9 females/6 males |
| Median symptom duration (range) | 7 months (2-12) |
| Ever smokers* | 3 |
| Prednisolone | 2/15 |
| NSAID | 11/15 |
| DAS28 (mean±SEM) | 5.6±0.2 |
| Median time to second biopsy (range) | 2 months (1.9-2.5) |
| Median time to clinical evaluation of response (range) | 3 months (2.5-4) |
| ACPA and/or RF positive | 9/15 |
| *5 missing data | |
