# Supplementary material for: Synovial membrane immunohistology in early-untreated rheumatoid arthritis reveals high expression of catabolic bone markers that is modulated by methotrexate
Source: Arthritis Res Ther. 2013 Dec 3;15(6):R205. doi: 10.1186/ar4398 (PMC3978873; doi:10.1186/ar4398)
Supplement: Additional file 2 — Presents supplementary methodological information. Description of the RANKL and OPG primer sequences. [file ar4398-S2.doc]

The following primers for RANKL detection in Saos2 cells for sense strand 5'-CCAAGATCTCCAACATGACT-3', RANKL anti-sense strand 5'-TACACCATTAGTTGAAGATACT-3', RANKL detection in RASF cells for sense strand 5'- ACCAGCATCAAAATCCCAAG-3', RANKL anti-sense strand 5'-CCCCAAAGTATGTTGCATCC-3', OPG sense strand 5'-AGGAAATGGCAACACACGACA-3', OPG anti-sense strand 5'-CCTGAAGAATGCCTCCTCAC-3', Ribosomal Protein L32 (RPL32) sense strand 5'-CAT CTC CTT CTC GGC ATC A-3', RPL32 anti-sense strand 5'- AAC CCT GTT GTC AAT GCC TC-3', Guanine Nucleotide Binding Protein (G-protein) beta Polypeptide 2-like 1 (GNB21) sense strand 5'-GAG TGT GGC CTT CTC CTC TG-3', GNB21 anti-sense strand 5'- GCT TGC AGT TAG CCA GGT TC-3'.
