# Supplementary material for: Synovial membrane immunohistology in early-untreated rheumatoid arthritis reveals high expression of catabolic bone markers that is modulated by methotrexate
Source: Arthritis Res Ther. 2013 Dec 3;15(6):R205. doi: 10.1186/ar4398 (PMC3978873; doi:10.1186/ar4398)
Supplement: Additional file 3 — Shows that MTX decreases mRNA and protein expression of RANKL and RANK in osteoblast-like tumoral cells. Graph showing a significant decrease in the RANKL mRNA levels with no changes in OPG mRNA levels in the presence of MTX (A). Representative blot and graphs showing decrease of cellular RANKL protein expression with no changes in the cellular OPG expression in the presence of MTX (B). Graphs showing decrease of soluble RANKL protein expression with no changes in the soluble OPG expression in the presence of MTX (C), where results are quantified by enzyme-linked immunosobent assay (ELISA). rtPCR, reverse transcriptase polymerase chain reaction. [file ar4398-S3.ppt]

## Slide 1
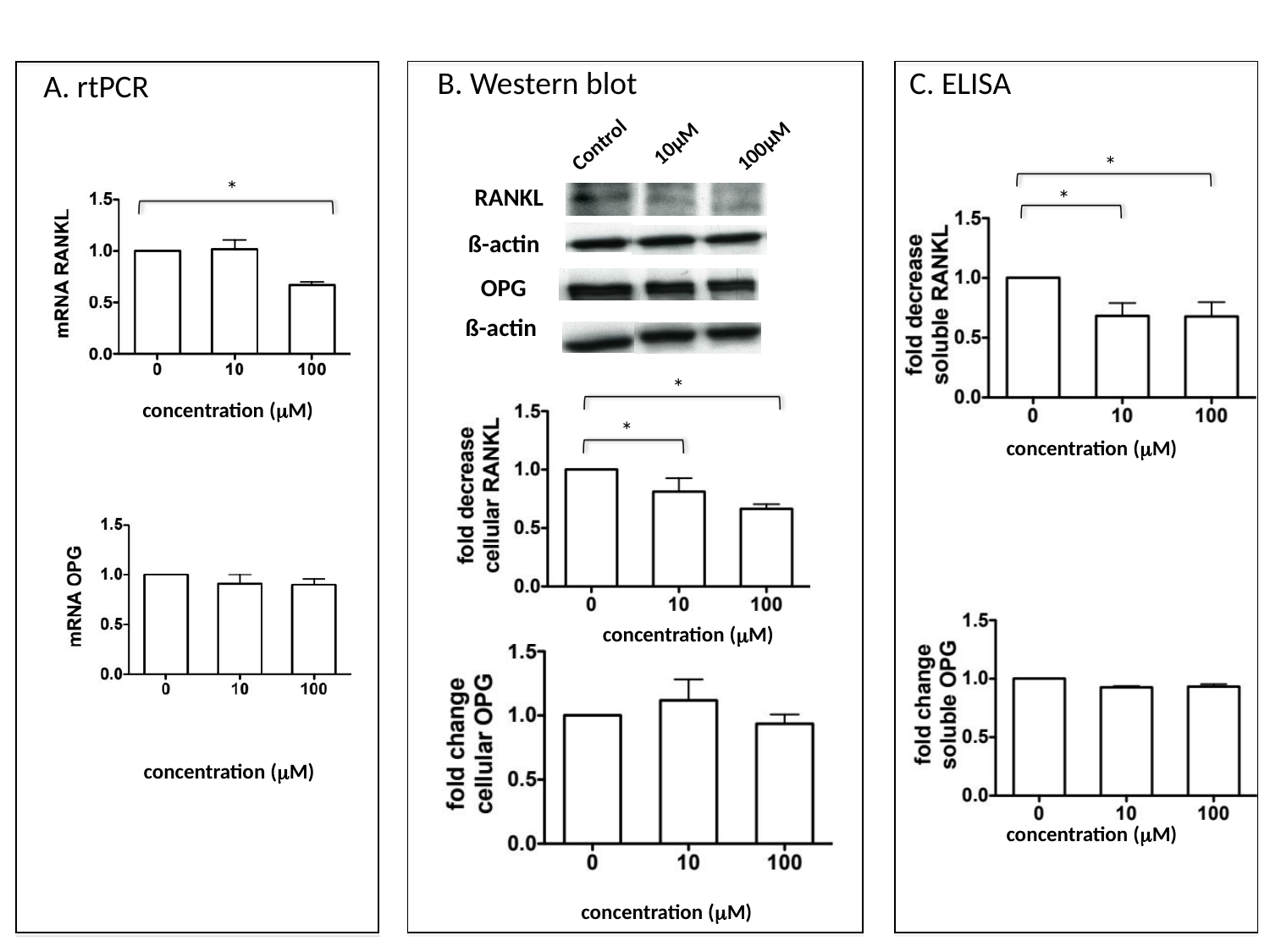

B. Western blot
C. ELISA
A. rtPCR
10µM
Control
100µM
*
*
 RANKL
*
 ß-actin
OPG
 ß-actin
*
concentration (μM)
*
concentration (μM)
concentration (μM)
concentration (μM)
concentration (μM)
concentration (μM)
