# Supplementary figures and images for: Synovial membrane immunohistology in early-untreated rheumatoid arthritis reveals high expression of catabolic bone markers that is modulated by methotrexate
Source: Arthritis Res Ther. 2013 Dec 3;15(6):R205. doi: 10.1186/ar4398 (PMC3978873; doi:10.1186/ar4398)

## Slide 1
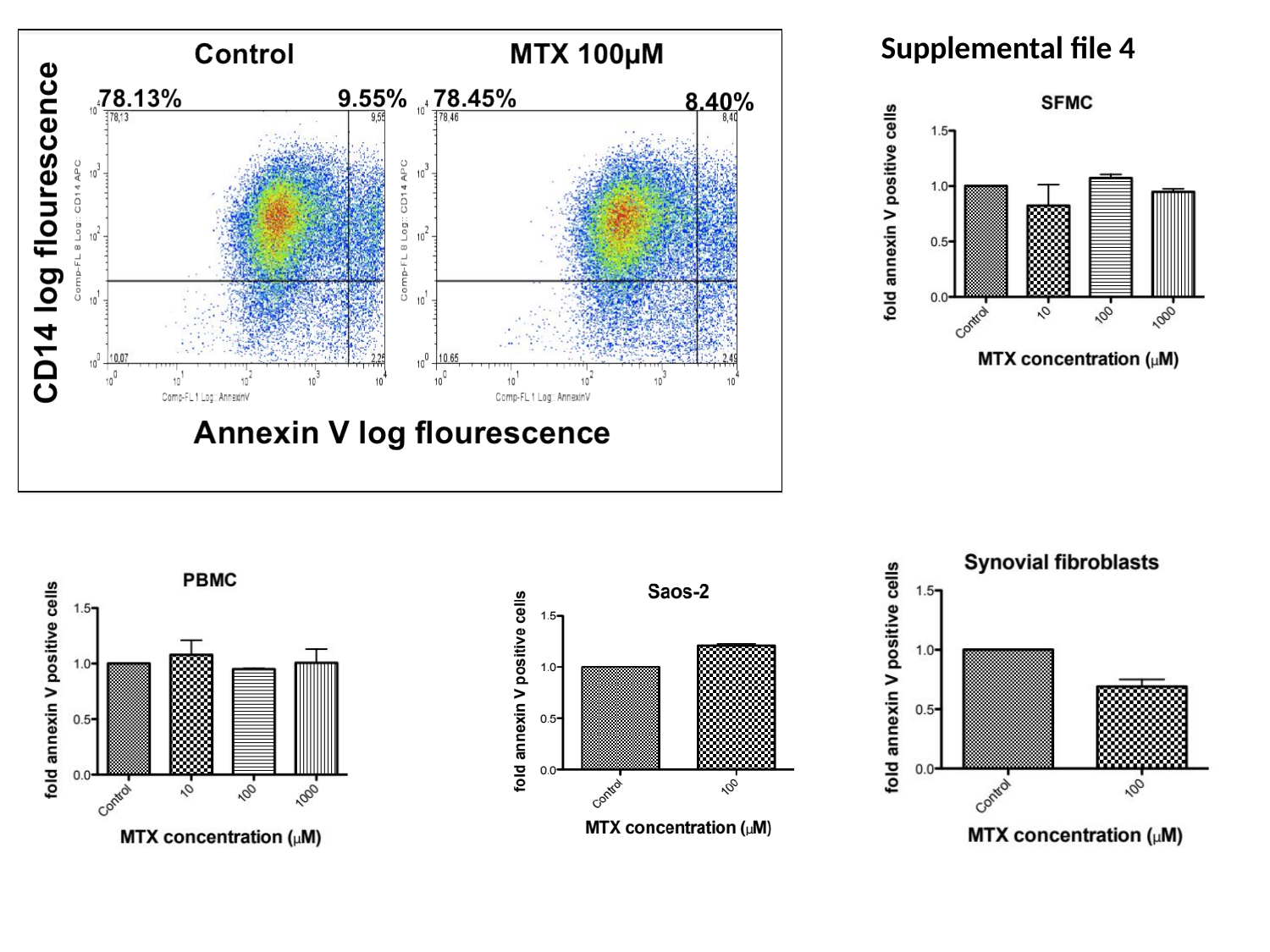

Supplemental file 4

Supplement: Additional file 4 — Shows the lack of apoptosis induction by MTX. Representative cytometry plots showing that MTX does not induce apoptosis and graphs representing quantification of Annexin-V-positive cells following MTX exposure in synovial fluid mononuclear cells (SFMC), PBMC, Saos-2 (osteoblast-like tumoral cells) and synovial fibroblasts. [file ar4398-S4.ppt]
